# Exploring ethnic differences in the perioperative course of thoracic aortopathy

**DOI:** 10.3389/fcvm.2025.1708865

**Published:** 2025-11-17

**Authors:** Nora Bacour, Aytug U. Tirpan, Simran Grewal, Robert J. M. Klautz, Nimrat Grewal

**Affiliations:** 1 Department of Cardiothoracic Surgery, Amsterdam University Medical Center Location AMC, Amsterdam, Netherlands; 2 Department of Orthopaedic Surgery, Onze Lieve Vrouwe Gasthuis, Amsterdam, Netherlands; 3 Department of Cardiothoracic Surgery, Leiden University Medical Center, Leiden, Netherlands; 4 Aortic Institute at Yale-New Haven Hospital, Yale University School of Medicine, New Haven, CT, United States

**Keywords:** aortopathy, ethnicity, health disparities, pre- and post-operative course, anxiety

## Abstract

**Background:**

In patients with thoracic aortopathy post-operative recovery can be complicated by a lack of consistent, clear communication and guidance. Despite the growing recognition of these challenges, the specific needs of patients undergoing aortic surgery, especially across different ethnic groups, remain insufficiently explored. This study aims to examine the peri-operative course in thoracic aortopathy in order to identify the specific needs of the patients, particularly across ethnic groups.

**Methods:**

This cross-sectional, retrospective study included patients who underwent aortic surgery between 2000 and 2023. Patients were asked to complete questionnaires assessing various parts of the pre-, peri-, and (long term) post-operative course. Results were compared between ethnic majority and ethnic minority patients, with further sub-categorization based on the type of aortopathy (aortic dissection vs. thoracic aortic aneurysm). Binary regression models were used, adjusted for age, sex, and follow-up duration when comparing aortopathy types, and additionally for aortic dissection diagnosis when comparing ethnic groups.

**Results:**

A total of 189 patients (174 ethnic majority, 15 minorities) were included. Of these, 115 had type A aortic dissection and 74 had a thoracic aortic aneurysm. Ethnic minority patients showed higher rates of post-operative anxiety (20.0% vs. 5.4%). Minority patients also reported less clarity in post-operative information (*p* = 0.035). Acute aortic dissection patients reported lower clarity of pre-operative information (7.0 vs. 9.0, *p* = 0.003) and worse understanding of recovery expectations before surgery (*p* < 0.001).

**Conclusion:**

This study highlights the critical importance of pre- and post-operative education in thoracic aortopathy patients. Ethnic disparities in recovery experiences exist, particularly in the domains of post-operative anxiety and communication. Our findings highlight the need for tailored post-operative guidance in order to improve recovery outcomes, and ensure more equitable care.

## 1 Introduction

Thoracic aortopathy encompasses various aortic diseases including a thoracic aortic aneurysm and aortic dissection (1). In patients with thoracic aortopathy life-saving surgical intervention is needed to reduce the high mortality rates (1). Prior research has demonstrated that patients who undergo aortic surgery frequently face significant challenges in their physical, mental, and emotional well-being post-operatively (2, 3). These challenges often require ongoing medical supervision and rehabilitation. However, post-operative recovery can be complicated by a lack of consistent, clear communication and guidance (4–6).

Given the complexity of thoracic aortopathy and the high-risk surgical intervention it entails, clear pre- and post-operative instructions are essential to maximise post-operative outcomes. In particular, a number of studies indicate that thorough pre-operative education regarding the procedure and any possible risks might help lower anxiety and improve the recovery trajectory (7–10). Without proper education on managing their recovery at home, patients may struggle to understand how to navigate the challenges of daily life, which can hinder their overall recovery trajectory.

Over the past decade, ethnic disparities in healthcare have become an increasingly important topic. Many studies have highlighted the impact of ethnicity on access to care, treatment outcomes, and post-operative recovery trajectories (11–13). While ethnicity is primarily a social construct encompassing shared origin, language, and cultural norms, belonging to an ethnic minority has been associated with disparities in healthcare experiences and outcomes (14). The leading hypothesis for these disparities is that they arise from a complex synergy of genetic predisposition, socioeconomic status, and healthcare access (15). Research has also shown that ethnic background can influence post-operative satisfaction in various surgical fields including orthopedic and bariatric surgery, particularly in terms of perceived recovery and rehabilitation outcomes (16, 17). Nonetheless, to our knowledge, studies on the pre- and post-operative course among ethnic groups with thoracic aortopathy have not yet been conducted.

Taken together, these findings raise important questions on pre- and post-operative experiences in thoracic aortopathy and how these may differ among ethnic populations undergoing thoracic aortic surgery. Therefore, in this study we aim to examine the pre- and post operative course in thoracic aortopathy in order to identify the specific needs of the patients. Additionally, we will investigate whether recovery experiences differ across ethnic groups to better support these patients. By identifying potential disparities in perioperative management and recovery, we seek to provide insights that may contribute to more personalized and equitable healthcare strategies.

## 2 Methods

### 2.1 Ethical considerations

The ethical standards set forth in the Declaration of Helsinki and the guidelines for Good Clinical Practice were followed in the conduct of this investigation, ensuring the confidentiality and anonymity of all collected data. The Medical Ethical Committee of the Leiden University Medical Center reviewed and approved our study protocol. Our study protocol was covered under the IHLCN biobank protocol, with assigned reference number: B21.051/MS/ms, and acceptance date 31-01-2024. Patients were asked to sign informed consent forms and could withdraw their participation at any given moment.

### 2.2 Patient selection

This study assessed the pre-, peri- and (long term) post-operative course among ethnic groups in aortopathy. Patients records from the Amsterdam University Medical Center (AUMC) and the Leiden University Medical Center (LUMC) between January 2000 to January 2023 were assessed and selected after surgical repair for a thoracic aortic aneurysm or a type a aortic dissection. These patients were identified and were contacted digitally to complete questionnaires designed to provide insights into their recovery experiences. Two reminders were sent, one and two weeks after the initial contact. Specific patient characteristics were retrospectively obtained through digital patient records, after inclusion in our study. Inclusion criteria were age >18 years and a confirmed diagnosis of aortopathy. There were no specific exclusion criteria besides not being able to complete the entire questionnaire.

### 2.3 Questionnaire

A questionnaire was developed to assess various aspects of the recovery period (Supplementary File I). The questionnaire was available in both Dutch and English.

#### 2.3.1 Patient demographics

Firstly, the questionnaire collected demographic patient data, including age, sex, weight in kg, height in cm, highest level of education and current employment. The ethnicity and gender of patients were based on recommendations for standardized collection of diversity data by the Joint Commitment for Action on Inclusion and Diversity in Publishing (18, 19). Patients who self-identified as non-white were classified as belonging to an ethnic minority group. (Supplementary File I).

#### 2.3.2 Anxiety

Following, the level of post-surgical anxiety was assessed using the Generalized anxiety disorder (GAD-7) questionnaire (20). This questionnaire contains seven questions. The presence of anxiety can be determined based on the responses to these questions. Each question is assigned a sub-score from 0–3, where 0 indicates not experiencing a certain complaint, and 3 indicates experiencing a certain complaint nearly every day. These seven sub-scores were then summed to form the GAD-7 total score, ranging from 0–21, with higher scores indicating a higher severity of generalized anxiety. Scores from 0–4 indicated the presence of minimal anxiety. Scores from 5–9 indicated mild anxiety. Scores from 10–14 indicated moderate anxiety. Lastly, scores from 15–21 indicated severe anxiety.

#### 2.3.3 Depression

Furthermore, the presence of depression at follow-up was assessed using the Patient Health Questionnaire (PHQ-9) questionnaire (21). This questionnaire consists of nine questions which determines the presence and severity of depression in patients. Similar to the previous questionnaire, the questions are all assigned a sub-score from 0–3, which similar ranking. These subscores were then also summed and the final scores, ranging from 0–27, were then used to determine the presence and severity of depression. Scores from 0–4 indicated the presence of minimal depression. Scores from 5–9 indicated mild depression. Scores from 10–14 indicated moderate depression. Scores from 15–19 indicated moderately severe depression. Lastly, scores from 20–27 indicated severe anxiety.

#### 2.3.4 Preoperative anxiety

Additionally, preoperative anxiety was assessed by asking patients to look back at the time before their surgical intervention and answer the questions from the Amsterdam Preoperative Anxiety and Information Scale (APAIS) questionnaire (22). Although this questionnaire is intended for use before surgery, in this study we applied it retrospectively to explore pre-operative anxiety, particularly among patients of ethnic minority descent, as previous research in this population is limited. This questionnaire consists of six statements that patients were asked to rate. This questionnaire assesses the presence of anxiety related to the surgical intervention that patients have had. Patients had to rate their agreement in regard to these statements from 1 (not at all), to 5 (extremely). To determine procedural anxiety the responses to statements 1, 2, 4 & 5 were summed. Scores from 0–9 indicated no procedural anxiety. Scores from 10–12 indicated the presence of procedural anxiety. Scores from 13–20 indicated high levels of procedural anxiety. To determine the need for information about the procedure, the responses to the statements 3 & 6 were summed. Scores from 0–4 indicated no need for information. Scores from 5–7 indicated that patients had an average need for information. Scores of 8–10 indicated a high need of information.

#### 2.3.5 Pre- and post-operative care assessment

Lastly, patients were asked to rate their received care across various domains on a scale from 1–10, with 1 being the lowest and 10 the highest. Additionally, they were asked about the way they were informed, their understanding of the recovery period before surgery, and the usefulness of the informational material they received. (Supplementary File I).

### 2.4 Retrospectively obtained data

In order to comprehensively understand the pre- and post-operative course in thoracic aortopathy, the questionnaire was complemented with patient characteristics at event, obtained from digital patient records. This data included whether patients had a thoracic aneurysm or a type A aortic dissection, the pre-operative weight in kg, height in cm, the aortic diameter at event, the aortic valve morphology, the occurrence of a complication during the recovery period, and lastly, the date of surgical intervention, in order to determine the follow-up time in years.

### 2.5 Statistical analysis

To summarize the baseline characteristics of post-dissection patients, descriptive statistics were employed. Variables were assessed in terms of distribution; normally distributed continuous data are presented as mean ± standard deviation (SD), whereas non-normally distributed continuous data are presented as median and interquartile range. Categorical data are presented as frequencies and proportions. Missing data was handled using complete case analysis. Primary outcomes of this study were patient-reported scores from validated questionnaires. Secondary outcomes included responses to questions on patient experience and overall satisfaction with the received care.

In addition to the comparison of ethnic minority groups in the Netherlands to the ethnic majority (European-Caucasian), disparities between patients with a thoracic aortic aneurysm and type A aortic dissection were also analyzed. To compare the findings between the groups we used a logistical regression model. A univariable approach was first used to assess all individual baseline characteristics. Following, we used a multivariable model when assessing the care evaluation. To compare patients with a thoracic aortic aneurysm to those with a type A aortic dissection, we adjusted for sex, age, and follow-up time. To compare ethnic majority patients to ethnic minority patients, we adjusted for age, sex, follow-up time, and diagnosis of a type A aortic dissection. A p-value of <0.05 was considered significant. All statistical analyses were conducted using IBM SPSS version 30.0.

## 3 Results

### 3.1 Study population

**Figure 1:**
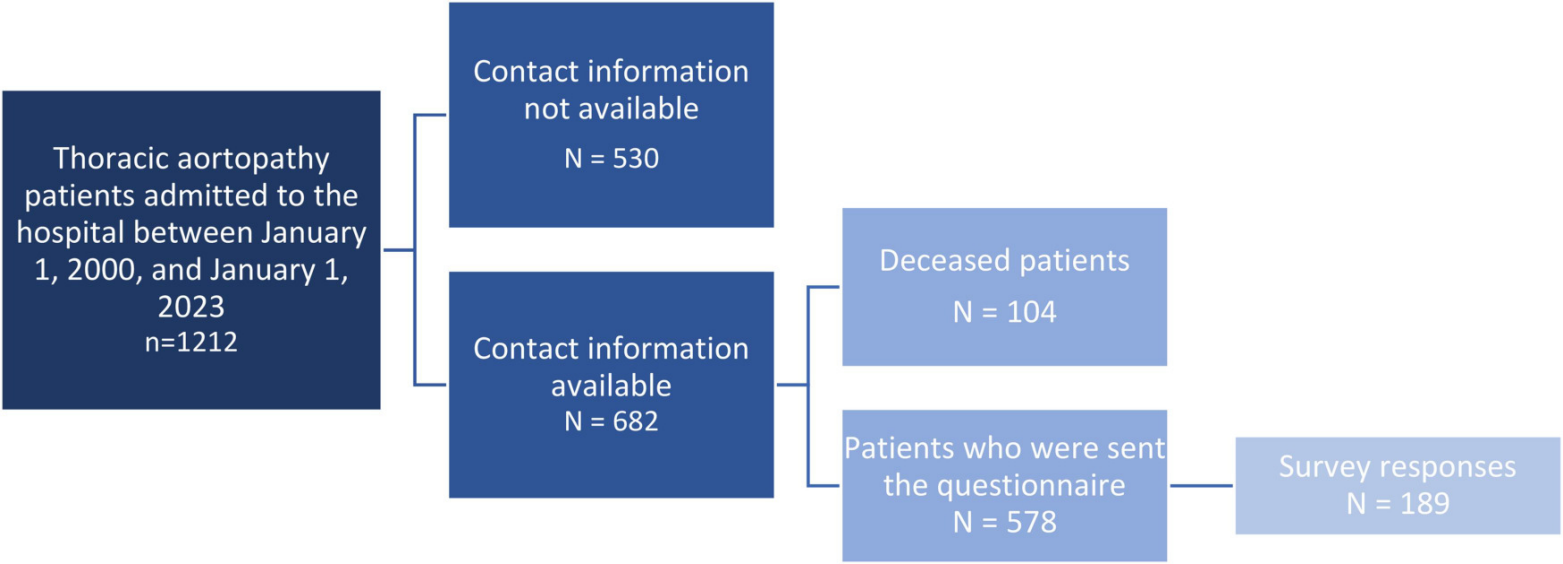
Sample selection. This figure shows a flow chart of inclusion in our study.

This study assessed the pre- and post-operative course among ethnic groups in aortopathy. Patients records from AUMC and the LUMC were studied between January 2000 to January 2023. These patients had all undergone surgical repair for a thoracic aneurysm or a type A aortic dissection. After initial screening, the study cohort included 1,212 patients. Of this cohort, contact information was only available in 682 patients. One hundred and four patients of this group were deceased, leaving 578 patients who received the request to complete the digital questionnaire. Survey responses were received from 189 patients, making the response rate 32.7% (Figure 1).

### 3.2 Pre-operative characteristics

**Figure 2:**
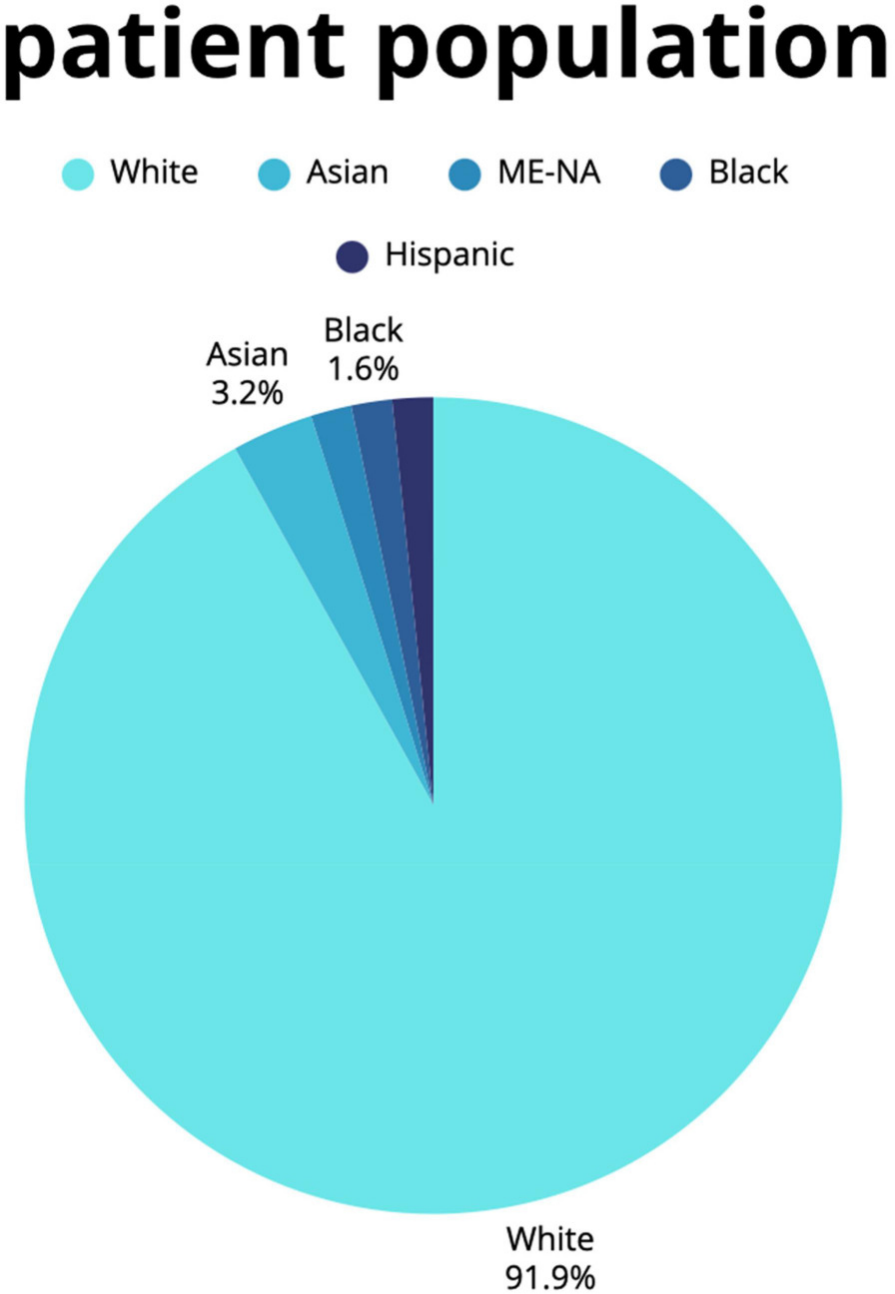
Patient population. This figure shows a pie chart of the proportion of racial backgrounds in our cohort.

Our study population, consisting of 189 patients was subdivided into an ethnic majority group (*n* = 174) and ethnic minority group (*n* = 15), which resembles the ethnic distribution of the Dutch population (23). Figure 2 shows the distribution of ethnic background of the patients within our cohort. Of the entire cohort, 115 patients (60.8%) had undergone surgical repair for a type A aortic dissection, and 74 patients (39.2%) had undergone repair for a thoracic aortic aneurysm. In the entire cohort of 189 patients, 44 patients (23.3%) were female and the mean age was 57.8 ± 10.9 at time of intervention. The BMI at event in patients with thoracic aortopathy was 26.6 ± 4.3. In terms of clinical presentation 52 patients (29.4%) presented with a bicuspid valve and the median aortic diameter was 49.0 (42.0–54.0). A bicuspid aortic valve was 6 times more prevalent in the aneurysm group as compared to the dissection patients (10.4% [aneurysm (a)] vs. 64.5% [dissection (d)]; OR 0.064 (0.029–0.142); *p* < 0.001). However, it should be noted that his percentage is relative as some missing values were present. Self-reported complications were found to be less prevalent in thoracic aneurysm patients compared to acute aortic dissection patients [48.6% (a) vs. 65.2% (d); OR 2.029 (1.111–3.705); *p* = 0.021]. A comprehensive comparison of the pre-operative characteristics in thoracic aortic aneurysm vs. acute type A aortic dissection and ethnic majority vs. ethnic minority can be found in Tables 1, 2, respectively.

**Table 1:** Patient characteristics among thoracic aortopathy.

Characteristic	Thoracic Aortic Aneurysm	Type A Aortic Dissection	OR (95 CI)	*P*-value
	*N* = 74	*N* = 115		
Patient characteristics at event				
Age	58.9 ± 10.9	57.2 ± 11.0	0.986 (0.959–1.013)	0.309
Weight (kg)	89.0 (76.0–98.0) (8)*	84.0 (75.0–95.0)	0.991 (0.972–1.010)	0.344
Height (cm)	180.9 ± 9.2 (8)*	180.4 ± 9.4 (1)*	0.994 (0.962–1.027)	0.727
BMI (kg/m^2^)	27.1 ± 4.5	26.3 ± 4.1 (1)*	0.956 (0.893–1.023)	0.195
Aortic Diameter	52.0 (48.0–55.0) (47)*	48.0 (41.0–54.0) (8)*	0.934 (0.887–0.984)	0.010
Bicuspid valve	40/62 (64.5%)	12/115 (10.4%)	0.064 (0.029–0.142)	<0.001
Complication during recovery	36/73 (48.6%)	75/113 (65.2%) (2)*	2.029 (1.111–3.705)	0.021
Patient characteristics at follow-up				
Age	68.2 ± 9.9 (3)*	64.8 ± 10.2 (5)*	0.966 (0.935–0.997)	0.031
Weight (kg)	87.0 (78.5–98.0) (1)*	84.0 (75.0–91.8) (3)*	0.986 (0.967–1.005)	0.153
Height (cm)	181.4 ± 9.1	179.8 ± 9.4	0.982 (0.952–1.014)	0.274
BMI (kg/m^2^)	26.9 ± 4.3 (1)*	26.3 ± 4.1 (3)*	0.965 (0.900–1.035)	0.317
Female	14/74 (18.9%)	30/115 (26.1%)	1.513 (0.740–3.093)	0.257
Follow-up (in years)	8.6 (5.9–11.6)	6.9 (4.1–8.9)	0.863 (0.798–0.934)	<0.001
Employment status				
Incapacitated Employed Retired Other	4/73 (5.6%)	23/114 (20.0%)	4.360 (1.441–13.189)	0.009
	22/73 (31.4%)	39/114 (33.9%)	1.205 (0.641–2.268)	0.562
	44/73 (62.9%)	49/114 (42.6%)	0.497 (0.273–0.903)	0.022
	4/73 (5.6%)	4/114 (3.5%)	0.631 (0.124–3.212)	0.579
Educational level				
Low Intermediate High	11/73 (15.1%)	11/114 (9.6%)	-	0.117
	25/73 (33.8%)	54/114 (46.9%)	2.160 (0.826–5.646)	0.116
	37/73 (50.0%)	49/114 (42.6%)	1.324 (0.518–3.385)	0.557

Data are presented as *N* (%), Mean ± SD or Median (IQR).

* Number of missing values are shown as (*n*)*.

**Table 2:** Patient characteristics among ethnic groups.

Characteristic	Total	Ethnic majority	Ethnic minority	OR (95 CI)	*P*-value
	*N* = 189	*N* = 174	*N* = 15		
Patient characteristics at event					
Age	57.8 ± 10.9	58.0 ± 10.8	55.9 ± 12.5	0.983 (0.938–1.030)	0.466
Weight (kg)	86.0 (75.0–96.0) (8)*	87.0 (77.0– 96.0) (7)*	75.0 (70.0–82.5) (1)*	0.966 (0.928–1.006)	0.099
Height (cm)	180.6 ± 9.3 (9)*	181.0 ± 9.1 (8)*	176.4 ± 10.4 (1)*	0.947 (0.892–1.006)	0.078
BMI (kg/m^2^)	26.6 ± 4.3 (1)*	26.7 ± 4.3 (1)*	25.6 ± 4.8	0.935 (0.815–1.073)	0.340
Aortic diameter	49.0 (42.0–54.0) (55)*	49.0 (43.0–54.0) (53)*	42.0 (38.5–51.0) (2)*	0.958 (0.907–1.013)	0.134
Bicuspid valve	52/177 (29.4%)	50/163 (30.7%)	2/14 (14.3%)	0.377 (0.081–1.746)	0.212
Aortic dissection	115/189 (60.8%)	103/174 (59.2%)	12/15 (80.0%)	2.757 (0.751–10.125)	0.126
Complication during recovery	111/186 (58.8%)	101/171 (59.1%)	10/15 (66.7%)	1.386 (0.454–4.231)	0.566
Patient characteristics at follow-up					
Age	66.1 ± 10.2 (8)*	66.4 ± 9.9 (8)*	63.4 ± 13.0	0.974 (0.928–1.022)	0.276
Weight (kg)	85.0 (75.0–94.5) (4)*	85.0 (77.0–95.0) (4)*	75.0 (70.0–94.0)	0.981 (0.945–1.019)	0.318
Height (cm)	180.4 ± 9.3	180.7 ± 9.2	177.4 ± 9.9	0.962 (0.908–1.020)	0.191
BMI (kg/m²)	26.5 ± 4.2 (4)*	26.6 ± 4.1 (4)*	26.2 ± 5.2	0.979 (0.860–1.115)	0.751
Female	44/189 (23.3%)	42/174 (24.1%)	2/15 (13.3%)	0.484 (0.105–2.230)	0.351
Follow-up (in years)	6.7 (4.9–10.0)	6.8 (4.9–10.1)	5.8 (3.5–7.1)	0.902 (0.770–1.057)	0.203
Employment status					
Incapacitated Employed Retired Other	27/187 (14.9%)	23/173 (13.3%)	4/14 (28.6%)	2.609 (0.755–9.013)	0.130
	61/187 (33.7%)	59/173 (34.1%)	2/14 (14.3%)	0.322 (0.070–1.487)	0.147
	93/187 (51.4%)	85/173 (49.1%)	8/14 (57.1%)	1.380 (0.460–4.146)	0.566
	8/187 (4.2%)	6/173 (3.5%)	0/14 (0.0%)	0.000 (0.000–0.000)	0.999
Educational level					
Low Intermediate High	22/187 (11.8%)	21/173 (12.1%)	1/14 (7.1%)	-	0.503
	79/187 (42.2%)	71/173 (41.0%)	8/14 (57.1%)	2.366 (0.280–20.013)	0.429
	86/187 (46.0%)	81/173 (46.8%)	5/14 (35.7%)	1.296 (0.144–11.699)	0.817

Data are presented as *N* (%), Mean ± SD or Median (IQR).

* Number of missing values are shown as (*n*)*.

### 3.3 Post-operative characteristics

At the time of follow up, the mean age was 66.1 ± 10.2 and the mean BMI was 26.5 ± 4.2. The median follow-up time was 6.7 (4.9–10.0) years, with a significantly longer follow-up in patients with thoracic aortic aneurysm patients compared to those with a type A aortic dissection [8.6 (a) vs. 6.9 (d) years; OR 0.863 (0.798–0.934); *p* < 0.001]. Overall, most patients were retired (93; 51.4%) and highly educated (86; 46.0%). When comparing thoracic aortic aneurysm patients to type A aortic dissection patients, we found that the aneurysm group had significantly more patients that were retired [62.9% (a) vs. 42.6% (d); OR 0.497 (0.273–0.903), *p* = 0.022] and the dissection group had higher levels of incapacitated patients [5.6% (a) vs. 20.0% (d); OR 4.360 (1.441–13.189), *p* = 0.009]. Additionally, no significant disparities were found between ethnic majority and ethnic minority patient groups (*p* > 0.05). A comprehensive comparison of the post-operative characteristics in thoracic aortic aneurysm vs. acute type A aortic dissection and ethnic majority vs. ethnic minority can again be found in Tables 1, 2, respectively.

### 3.4 Depression and (pre-operative) anxiety

Depression severity, as assessed using the PHQ-9 questionnaire (21) showed that 15 patients (7.9%) had moderate to severe prevalence of depression. When comparing thoracic aortic aneurysms to acute type A aortic dissection and ethnic majority patients to ethnic minority patients, these proportions were not significant (*p* > 0.05) (Tables 3, 4).

**Table 3:** Multivariable regression model for care evaluation in thoracic aortopathy.

Characteristic	Thoracic Aortic Aneurysm	Type A Aortic Dissection	OR (95 CI)	*P*-value
	*N* = 74	*N* = 115		
GAD-7: Anxiety severity				
Minimal anxiety Mild anxiety Moderate anxiety Severe anxiety	59/70 (84.3%)	90/113 (79.6%)	-	0.642
	8/70 (11.4%)	14/113 (12.4%)	1.093 (0.397–3.012)	0.864
	1/70 (1.4%)	7/113 (6.2%)	3.781 (0.431–33.156)	0.230
	2/70 (2.9%)	2/113 (1.8%)	0.608 (0.068–5.437)	0.657
PHQ-9: Depression severity				
Minimal depression Mild depression Moderate depression Moderately severe depression Severe depression	60/74 (81.1%)	83/115 (72.2%)	-	0.856
	11/74 (14.9%)	20/115 (17.4%)	1.102 (0.448–2.710)	0.832
	2/74 (2.7%)	10/115 (8.7%)	2.260 (0.423–12.088)	0.340
	1/74 (1.4%)	1/115 (0.9%)	0.416 (0.024–7.330)	0.549
	0/74 (0.0%)	1/115 (0.9%)	0.000 (0.000–0.000)	1.000
Amsterdam Preoperative Anxiety and Information Scale				
Information desire component				
No need for information Need for information Severe need for information	43/71 (60.6%)	94/111 (84.7%)	-	<0.001
	22/71 (31.0%)	12/111 (10.8%)	0.180 (0.073–0.447)	<0.001
	6/71 (8.5%)	5/111 (4.5%)	0.438 (0.119–1.618)	0.216
Combined anxiety component				
No procedural anxiety Procedural anxiety Severe procedural anxiety	60/71 (84.5%)	102/111 (91.9%)	-	0.029
	8/71 (11.3%)	2/111 (1.8%)	0.112 (0.022–0.561)	0.008
	3/71 (4.2%)	7/111 (6.3%)	0.864 (0.184–4.053)	0.853
Pre- and post-operative care assessment				
How were patients informed?				
Not informed pre-operatively Verbal Written Post-op patient information session	16/74 (21.7%)	66/115 (57.4%)	4.052 (1.985–8.272)	<0.001
	47/74 (63.5%)	45/115 (39.1%)	0.424 (0.219–0.821)	0.011
	6/74 (8.1%)	2/115 (1.7%)	0.221 (0.042–1.173)	0.076
	5/74 (6.8%)	2/115 (1.7%)	0.358 (0.064–2.018)	0.244
Care assessment scale				
Clarity of pre-operative information Recovery vs. expectations Clarity of post-operative information Satisfaction with communication with healthcare providers	9.0 (8.0–9.0)	7.0 (2.0–9.0)	0.828 (0.731–0.938)	0.003
	8.0 (4.0–9.0)	6.0 (5.0–8.0)	0.984 (0.871–1.112)	0.796
	8.0 (7.0–9.0)	8.0 (7.0–8.0)	1.170 (1.001–1.367)	0.048
	8.0 (7.0–9.0)	8.0 (7.0–9.0)	0.075 (0.910–1.271)	0.394
Usefulness of informational material				
Useful Unuseful	56/72 (77.8%)	67/111 (60.4%)	0.472 (0.225–0.989)	0.047
	16/72 (22.2%)	44/111 (39.6%)	-	
Understanding of recovery before surgery				
Clear Unclear	38/73 (52.1%)	20/112 (17.9%)	-	<0.001
	35/73 (47.9%)	92/112 (82.1%)	0.277 (0.135–0.566)	
Influence of race/ethnicity on received care				
Perceived relevance of ethnicity/origin questions to care				
Not important Important Not sure	48/73 (65.8%)	72/113 (63.7%)	-	0.691
	10/73 (13.7%)	12/113 (10.6%)	0.648 (0.228–1.843)	0.416
	15/73 (20.5%)	29/113 (25.7%)	1.036 (0.469–2.291)	0.913
My race/ethnicity impacted the care I received				
Yes No	4/74 (5.4%)	2/112 (1.8%)	0.324 (0.053–1.999)	0.225
	70/74 (94.6%)	110/112 (98.2%)	-	

Multivariable regression model corrected for age, sex, and follow-up time.

* Relative percentage due to missing values.

**Table 4:** Multivariable regression model for care evaluation among ethnic groups.

Characteristic	Total	Ethnic majority	Ethnic minority	OR (95 CI)	*P*-value
	*N* = 189	*N* = 174	*N* = 15		
GAD-7: Anxiety severity					
Minimal anxiety Mild anxiety Moderate anxiety Severe anxiety	149/183 (81.4%)	139/168 (82.7%)	10/15 (66.7%)	-	0.169
	22/183 (12.0%)	20/168 (11.9%)	2/15 (13.3%)	1.523 (0.302–7.672)	0.610
	8/183 (4.4%)	5/168 (3.0%)	3/15 (20.0%)	6.607 (1.512–34.517)	0.025
	4/183 (2.2%)	4/168 (2.4%)	0/15 (0.0%)	0.000 (0.000–0.000)	0.999
PHQ-9: Depression severity					
Minimal depression Mild depression Moderate depression Moderately severe depression Severe depression	143/189 (75.7%)	133/174 (76.4%)	10/15 (66.7%)	-	0.719
	31/189 (16.4%)	26/174 (14.9%)	5/15 (33.3%)	2.462 (0.726–8.352)	0.148
	12/189 (6.3%)	12/174 (6.9%)	0/15 (0.0%)	0.000 (0.000–0.000)	0.999
	2/189 (1.1%)	2/174 (1.1%)	0/15 (0.0%)	0.000 (0.000–0.000)	0.999
	1/189 (0.5%)	1/174 (0.6%)	0/15 (0.0%)	0.000 (0.000–0.000)	1.000
Amsterdam Preoperative Anxiety and Information Scale					
Information desire component					
No need for information Need for information Severe need for information	137/182 (75.3%)	124/168 (73.8%)	13/14 (92.9%)	-	0.495
	34/182 (18.7%)	32/168 (19.6%)	1/14 (7.1%)	0.269 (0.031–2.357)	0.236
	11/182 (6.0%)	11/168 (6.5%)	0/14 (0.0%)	0.000 (0.000–0.000)	0.999
Combined procedural anxiety component					
No procedural anxiety Procedural anxiety Severe procedural anxiety	162/182 (89.0%)	149/168 (88.7%)	13/14 (92.9%)	-	0.999
	10/182 (5.5%)	10/168 (6.0%)	0/14 (0.0%)	0.000 (0.000–0.000)	0.999
	10/182 (5.5%)	9/168 (5.4%)	1/14 (7.1%)	0.096 (0.096–9.259)	0.960
Pre- and post-operative care assessment					
How were patients primarily informed?					
Not informed pre-operatively Verbal Written Post-op patient information session	82/189 (43.4%)	79/174 (45.4%)	5/15 (33.3%)	0.452 (0.138–1.481)	0.190
	92/189 (48.7%)	82/174 (47.1%)	10/15 (66.7%)	2.806 (0.876–9.990)	0.083
	8/189 (4.2%)	8/174 (4.6%)	0/15 (0.0%)	0.000 (0.000–0.000)	0.999
	7/189 (3.7%)	7/174 (4.0%)	0/15 (0.0%)	0.000 (0.000–0.000)	0.999
Care assessment rating (from 1 to 10)					
Clarity of pre-operative information Recovery vs. expectations Clarity of post-operative information Satisfaction with communication with healthcare providers	8.0 (5.0–9.0)	8.0 (5.0–9.0)	9.0 (5.8–9.0)	0.223 (0.027–1.858)	0.056
	7.0 (5.0–8.0)	7.0 (5.0–8.0)	6.0 (2.0–8.3)	0.920 (0.746–1.134)	0.433
	8.0 (7.0–9.0)	8.0 (7.0–9.0)	7.0 (2.0–8.0)	0.772 (0.602–0.982)	0.035
	8.0 (7.0–9.0)	8.0 (7.0–9.0)	7.0 (6.0–9.0)	0.854 (0.658–1.108)	0.235
Usefulness of informational material					
Useful Unuseful	123/183 (67.2%)	113/169 (66.9%)	10/15 (71.4%)	1.231 (0.350–4.330)	0.746
	60/183 (32.8%)	56/169 (33.1%)	4/15 (28.6%)	-	
Understanding of recovery before surgery					
Clear Unclear	58/185 (31.4%)	54/170 (31.8%)	4/15 (26.7%)	1.271 (0.342–4.722)	0.720
	127/185 (68.6%)	116/170 (68.2%)	11/15 (73.3%)	-	
Influence of race/ethnicity on received care					
Perceived relevance of ethnicity/origin questions to care					
Not important Important Not sure	120/186 (64.5%)	109/171 (63.7%)	11/15 (73.3%)	-	0.500
	22/186 (11.8%)	20/171 (11.7%)	2/15 (13.3%)	1.228 (0.230–6.558)	0.810
	44/186 (23.7%)	42/171 (24.6%)	2/15 (13.3%)	0.410 (0.084–2.008)	0.272
My race/ethnicity impacted the care I received					
Yes No	6/186 (3.2%)	5/171 (2.9%)	1/15 (6.7%)	4.443 (0.408–48.413)	0.221
	180/186 (95.2%)	166/171 (95.4%)	14/15 (93.3%)	-	

Multivariable regression model corrected for age, sex, diagnosis of ATAAD and follow-up time.

Data are presented as N (%).

* Relative percentage due to missing values .

Following, the presence of preoperative anxiety, as assessed using the APAIS questionnaire (22), showed that 45 patients (24.7%) of patients desired information pre-operatively. When comparing thoracic aortic aneurysms to acute type A aortic dissection we found that patients with thoracic aortic aneurysms were significantly more likely to report a general need for information compared with those with acute type A aortic dissection [31.0% (a) vs. 10.8% (d); OR 0.180 (0.073–0.447), *p* < 0.001]. However, the proportion of patients reporting a severe need for information did not differ significantly between groups [8.5% (a) vs. 4.5% (d); OR 0.438 (0.119–1.618), *p* = 0.216]. Nevertheless, following survey distribution, we received feedback from several ATAAD patients indicating they responded neutrally to the information-related items, due to the absence of a pre-operative phase. This may partially account for the significantly lower reported desire for information in this group. These disparities were not found in ethnic majority patients compared ethnic minority patients (*p* > 0.05).

Procedural anxiety was found in 20 patients (11.0%). When comparing thoracic aortic aneurysms to acute type A aortic dissection, procedural anxiety was found in 11 patients (15.5%) compared to 9 patients (8.1%) respectively. Comparable to the findings on information desire, a significant difference was also observed in general procedural anxiety, which was more prevalent among patients with thoracic aortic aneurysm patients than in those with acute type A aortic dissections [11.3% (a) vs. 1.8% (d); 0.112 (0.022–0.561), *p* = 0.008]. In contrast, the proportion of patients reporting severe procedural anxiety did not differ significantly between the groups [4.2% (a) vs. 6.3% (d); 0.924 (0.198–4.315), *p* = 0.920] (Tables 3, 4).

Lastly, generalized anxiety, as assessed using the GAD-7 questionnaire (20) showed that moderate to high anxiety rates were seen in 12 patients post-operatively (6.6%). When comparing thoracic aortic aneurysms to acute type A aortic dissection no significant disparities were found (*p* > 0.05). However, moderate anxiety was significantly more prevalent in ethnic minority patients compared to ethnic majority patients, even after adjusting for age, sex, type of aortopathy diagnosis and follow up duration [3.0% (ethnic majority) vs. 20.0% (ethnic minority); OR 6.607 (1.512–34.517), *p* = 0.025] (Tables 3, 4).

### 3.5 Pre- and post-operative care assessment

Our questionnaire assessed various aspects of the pre- and post-operative course in patients with thoracic aortopathy. First, we examined how patients received information. In the overall population, as well as in comparisons between ethnic majority and ethnic minority patients, the primary mode of information delivery was similar, with verbal communication being the most commonly reported method. However, when comparing thoracic aortic aneurysms patients to those with an acute type A aortic dissection, significant disparities were found. Most patients with an acute type A aortic dissection were not informed pre-operatively [21.7% (a) vs. 57.4% (d); OR 4.052 (1.985–8.272), *p* < 0.001], whereas most patients with thoracic aortic aneurysms were informed verbally [63.5% (a) vs. 39.1% (d); OR 0.424 (0.219–0.821), *p* = 0.011] (Tables 3, 4).

Furthermore, we asked patients to rate various parts of the received care from 1–10. When comparing thoracic aortic aneurysms to acute type A aortic dissection we found that patients with acute type A aortic dissections reported lower median ratings of the clarity of pre-operative information when being compared to the thoracic aneurysm group [9.0 (a) vs. 7.0 (d); OR 0.828 (0.731–0.938), *p* = 0.003]. Additionally, patients belonging to an ethnic minority group reported lower median ratings of the clarity of post-operative information compared to ethnic majority patients [8.0 (ethnic majority) vs. 7.0 (ethnic minority); OR 0.772 (0.602–0.982), *p* = 0.035] (Tables 3, 4).

Lastly, we asked patients if they felt that their race/ethnicity impacted their received care. In general, 180 patients (95.2%) did not find that their ethnic origin impacted the care they received. This finding did not significantly differ among patients of ethnic minority descent (*p* > 0.05). Additionally, we also asked patients to give their opinion on whether the patients found questions on race and ethnicity in regard to medical care relevant. In general, the majority of patients found ethnic information not important [120 patients (64.5%)]. Slightly higher proportions of ethnic minority patients did find this to be important, while this disparity was not significant [11.7% (ethnic majority) vs. 13.3% (ethnic minority); OR 1.228 (0.230–6.558), *p* = 0.810] (Tables 3, 4).

### 3.6 Patient recommendations on improving information provision

The usefulness of the information provided to patients was classified by these patients as either “useful” or “not useful”. The majority of patients [123 (67.2%)] found the informational material to be useful. When comparing patients with a thoracic aortic aneurysm to those with an aortic dissection, the proportion of patients finding the material unhelpful differed significantly; less patients with an aortic dissection found the informational material useful compared to patients with a thoracic aortic aneurysm [77.8% (a) vs. 60.4% (d); OR 0.472 (0.225–0.989), *p* = 0.047] (Tables 3, 4).

Additionally, patients were asked about their clarity of understanding regarding the recovery process before surgery. In our entire study cohort, 127 patients (68.6%) reported that they had an unclear understanding of the post-operative recovery period before it began. This was particularly evident in patients with an acute type A aortic dissection, as compared to those with an aortic aneurysm [47.9% (a) vs. 82.1% (d); 0.277 (0.135–0.566), *p* < 0.001] (Tables 3, 4).

In the last segment of our questionnaire, we gave patients the opportunity to give their opinion on how to best enhance information provision (Tables 3, 4). Among the proposed improvements were:

1. Enhancement of (general) information provision (*n* = 16)
  - Use clear and simple language in pre- and post-operative information (*n* = 8)
  - Provide booklets with general information (*n* = 1)
  - Make more online information available (*n* = 1)
  - Use video materials for clarification (*n* = 4)
  - Provide alternative forms of information (*n* = 1)
  - Offer concise information (*n* = 1)
2. Better post-operative support (*n* = 13)
  - Personalized post-operative guidance, especially in case of complications (*n* = 6)
  - Mental support and assistance in processing experiences (e.g., PTSD) (*n* = 4)
  - Clear explanation of possible residual symptoms (*n* = 3)
3. Improved communication and coordination (*n* = 5)
  - Better communication between doctors and nursing staff (*n* = 1)
  - Clear identification of contact persons for patients (*n* = 1)
  - One-on-one discussions covering the entire process and possible scenarios (*n* = 1)
  - Consultation with an experienced physician instead of residents (*n* = 2)
4. More personalized and targeted post-operative support (*n* = 9)
  - Personal advice tailored to the patient (*n* = 5)
  - Involvement of the patient's partner in the process (*n* = 3)
  - Opportunity to ask medical questions (*n* = 1)
5. Better preparation for surgery (*n* = 9)
  - Preoperative consultation with an experienced physician (*n* = 1)
  - Clear explanation of check-ups and what the patient can expect (*n* = 8)

## 4 Discussion

This study aimed to examine the pre- and post-operative course in thoracic aortopathy. Specifically, we investigated whether recovery experiences differed across ethnic groups.

### 4.1 Depression and (pre-operative) anxiety

One of the key findings from this study was that overall anxiety rates were found to be relatively more prevalent compared to normative values. Previous research reported moderate to severe anxiety in 5.0% of a normative sample (Germany) (24). Our study showed higher anxiety rates than the average in both the ethnic majority and ethnic minority groups, as well as in patients with an aortic dissection, but not in those with a thoracic aortic aneurysm. The finding of heightened anxiety rates in type A aortic dissection was comparable to previous studies (2, 3, 25, 26). A study by McEntire et al. (27) showed that psychological distress can arise due to severe physical inactivity. This may be particularly relevant for aortopathy patients who experience significant physical activity restrictions, leading to reduced functional capacity and quality of life, which in turn may negatively impact their mental well-being (28). Previous research has demonstrated that physical activity influences brain structure and function; for example, it reduces levels of BDNF (brain-derived neurotrophic factor), which has been associated with increased anxiety and depression. At the same time, physical activity boosts dopamine, serotonin, and noradrenaline levels, which offer protective effects against the development of mental health disorders (29–31). Also, in case of pre-operative anxiety in elective aortic surgery, this can be improved by stimulating physical activity, which in turn can enhance the recovery trajectory (32). Therefore, encouraging physical activity is crucial in this patient group to mitigate these psychological issues.

Post-operative anxiety was also elevated in ethnic minority patients, even after adjusting for age, sex, follow-up duration and diagnosis of a type A aortic dissection. This is particularly interesting as, to our knowledge, previous research has not yet identified this trend in aortic surgery patients. Previous research has shown that this population often faces differences in cultural perceptions of illness, socioeconomic status, communication barriers between patients and healthcare providers, and variations in disease presentation or access to healthcare services (33–35). These factors may contribute to the development of post-operative anxiety in this group. However, further investigation into the psychosocial factors contributing to ethnic differences in anxiety and depression would be valuable, to better understand the mechanisms driving these disparities.

In patients with thoracic aortic aneurysms, the prevalence of procedural anxiety was significantly higher than in patients with aortic dissection, contrary to our hypothesis. However, as stated in the results, some patients with aortic dissections reported providing neutral responses regarding their pre-operative experience, as not all of them consciously remembered their pre-operative phase. Consequently, these results should be considered exploratory. The APAIS questionnaire should ideally be administered pre-operatively, but since we lacked such data, we attempted to approximate this time period in our analysis.

To obtain more accurate results, a prospective comparison is needed in a future study, as our current findings may underestimate anxiety levels and the need for information in this field. However, the overall demand for information was high across all subgroups, particularly among thoracic aneurysm patients. Considering these findings, it is evident that there is a significant need for information within our patient population.

On the contrary, overall depression rates were relatively low across all subgroups when compared to normative values. Previous research has found moderate to severe depression in 5.6% of a normative sample (Germany) (36). Our study showed lower depression rates than this average. Other research also observed these trends, indicating that depression rates were comparable to those of patients without thoracic aortopathy (37, 38).

### 4.2 Pre- and post-operative care assessment

The post-operative care experience was also evaluated, and it was found that most patients received verbal information. Previous research indicates that direct communication with patients can improve individualized care, is more specific than educational materials, and can help reduce procedural anxiety (8). However, we still found suboptimal scores in the ratings of pre- and post-operative information clarity. In regard to ethnic based disparities, patients themselves did not report that their ethnicity significantly influenced their received care or impacted their treatment experience. However, as previously mentioned, the results on quality assessments and anxiety rates hint at the possibility of differences in how information was received or processed by some patient groups. This indicates room for improvement in pre-operative education, particularly for these patient groups.

Several studies suggest that this could be addressed through a health promotion program that considers all patient needs both pre- and post-operatively (39, 40). We suggest, however, that such programs should be developed in close collaboration with the target population to meet the broad needs of all individuals. This, in turn, can enhance patient engagement and satisfaction, leading to improved health outcomes. For instance, a review by Stenberg et al. (41) highlighted that co-created group-based patient education programs, involving both healthcare professionals and patients from the target group, effectively promote self-management, which can enable patients to actively work towards a healthy post-operative life. Furthermore, the World Health Organization emphasized that therapeutic patient education should be tailored to the individual needs of patients, suggesting that collaborative development ensures relevance and effectiveness (42).

### 4.3 Patient recommendations on improving information provision

Patients provided valuable feedback on how to improve information provision and overall care. Most patients recommended: enhancement of (general) information provision, better post-operative support, improved communication and coordination, more personalized and targeted post-operative support, and better preparation for surgery. Adding to these valuable insights, previous research suggests that standardized information provision is often lacking, yet could be highly beneficial (43). Optimizing pre-operative education can significantly enhance overall patient satisfaction while reducing psychological stress and post-operative anxiety, particularly in cardiac surgery patients (43, 44).

### 4.4 Limitations

It is important to acknowledge the limitations of our study. Firstly, our study was conducted among patients who underwent surgery between 2000 and 2023, resulting in significant disparities in follow-up time. While we did adjust for follow-up duration in our multivariate analysis, this could potentially affect our findings. Following, as participation in our study was voluntary and limited to those who responded to our questionnaire invitation, this may have introduced some selection bias. However, this also presents a learning opportunity; we could prospectively ask patients to complete our questionnaires at specific time points, allowing for broader generalizability. Additionally, our small sample size could have influenced our results. At the same time, we are studying rare diseases, which inherently leads to smaller sample sizes, as seen in other studies (3, 6).

## 5 Conclusion

In conclusion, this study highlights the critical importance of pre- and post-operative education in thoracic aortopathy patients and suggests that ethnic disparities in recovery experiences exist, particularly in the domains of post-operative anxiety and communication. The findings call for more personalized, culturally sensitive care models that address the specific needs of diverse patient populations. By improving communication and providing more tailored information, healthcare providers can help reduce anxiety, improve recovery outcomes, and ensure more equitable care for all patients undergoing thoracic aortic surgery.

## Data Availability

The raw data supporting the conclusions of this article will be made available by the authors, without undue reservation.
