# Supplementary material for: Exploring ethnic differences in the perioperative course of thoracic aortopathy
Source: Front Cardiovasc Med. 2025 Nov 17;12:1708865. doi: 10.3389/fcvm.2025.1708865 (PMC12665577; doi:10.3389/fcvm.2025.1708865)
Supplement: Supplementary file 1 [file Datasheet1.pdf]

Supplementary material:
Supplement I: Questionnaire

Personal information

1. What is your date of birth?
2. What is your gender?
    - Female
    - Male
    - Transgender
    - Non-binary/Gender non-conforming
    - I prefer not to answer
3. What is your weight in kilograms?
4. What is your height in centimeters?

Questions Related to Ethnicity

The following questions pertain to your ethnic background. With this survey, we aim to understand the role of ethnic background in the experience of medical care.

5. What is your nationality?
    - Dutch
    - Other, namely: _______
6. Were you born in the Netherlands?
    - Yes
    - No (please specify): _______
7. What is your ethnic origin? Select all geographic areas where your ancestors are originally from, and explain the proportion of each origin in the text box:
    - Western Europe (e.g., Netherlands, United Kingdom)
    - Eastern Europe (e.g., Hungary, Poland, Russia)
    - North Africa (e.g., Egypt, Morocco, Sudan)
    - Sub-Saharan Africa (e.g., Kenya, Nigeria, South Africa)
    - West Asia / Middle East (e.g., Turkey, Iran, Saudi Arabia)
    - South and Southeast Asia (e.g., India, Indonesia, Singapore)
    - East and Central Asia (e.g., China, Japan, Uzbekistan)
    - Pacific/Oceania (e.g., Australia, Fiji, Papua New Guinea)
    - North America (e.g., Canada, United States)
    - Central America and the Caribbean (e.g., Jamaica, Mexico, Panama)
    - South America (e.g., Brazil, Chile, Colombia)
    - I prefer not to answer
    - Multiple ethnicities/Other (please specify): _______
8. How would you identify yourself in terms of race? Select all groups that apply to you, and describe the proportion in the text box:
    - Asian or Pacific Islander
    - Black
    - Hispanic or Latino/a/x
    - Indigenous (e.g., North American Indian Navajo, South American Indian Quechua, Aboriginal or Torres Strait Islander)
    - Middle Eastern or North African
    - White
    - I don’t know
    - I prefer not to answer
    - Multiple ethnicities/Other (please specify): _______

9. What ethnicity(s)/origin(s) describe your biological mother?
    - [Same options as Question 7]
10. What ethnicity(s)/origin(s) describe your biological father?

- [Same options as Question 7]

11. What ethnicity(s)/origin(s) describe your adoptive parents/guardian?

- Not applicable
- [Same options as Question 7]

Education and Employment

12. What is your highest level of education completed?

- Low (primary education, VMBO, MBO-1, first three years of HAVO/VWO)
- Intermediate (HAVO/VWO, MBO-2 to MBO-4)
- High (HBO or university [WO])

13. Employment status:

- Employed
- Retired
- Unable to work due to disability
- Other (please specify): _______

(Pre-operative) Anxiety and Depression

14. In the past 2 weeks, how often have you experienced one or more of the following problems?

**Problem**	Not at all	Some days	More than half of all days	Nearly every day
Feeling nervous, uneasy, or tense	☐	☐	☐	☐
Unable to stop worrying or unable to control worrying	☐	☐	☐	☐
Worrying too much about different things	☐	☐	☐	☐
Difficulty relaxing	☐	☐	☐	☐
Feeling so restless that sitting still was hard	☐	☐	☐	☐
Being easily irritated or annoyed	☐	☐	☐	☐
Feeling fearful as if something terrible might happen	☐	☐	☐	☐
Feeling nervous, uneasy, or tense	☐	☐	☐	☐
Unable to stop worrying or unable to control worrying	☐	☐	☐	☐

15. **I**n the past 2 weeks, how often have you experienced one or more of the following problems?

Problem	Not at all	Some days	More than half of all days	Nearly every day
Little interest or pleasure in activities	☐	☐	☐	☐
Feeling down, depressed, or hopeless	☐	☐	☐	☐
Difficulty falling asleep, staying asleep, or sleeping too much	☐	☐	☐	☐
Feeling tired or having a lack of energy	☐	☐	☐	☐
Poor appetite or overeating	☐	☐	☐	☐
Feeling bad about yourself, feeling like a failure, or that you let yourself or your family down	☐	☐	☐	☐
Trouble concentrating (e.g., reading the newspaper or watching TV)	☐	☐	☐	☐

16. **A**nswer the following questions based on how you felt before the operation:

Question (1 = Not at all, 5= Extremely)	1	2	3	4	5
I worry about the anesthesia	☐	☐	☐	☐	☐
The anesthesia constantly occupies my thoughts	☐	☐	☐	☐	☐
I would like to know as much as possible about the anesthesia	☐	☐	☐	☐	☐
I worry about the procedure/operation	☐	☐	☐	☐	☐
The procedure/operation constantly occupies my thoughts	☐	☐	☐	☐	☐

Question (1 = Not at all, 5= Extremely)	1	2	3	4	5
I would like to know as much as possible about the procedure/operation	☐	☐	☐	☐	☐

Surgical Experience

17. How were you informed about the operation?

- Verbal explanation
- Written information
- Informational meeting
- Online message
- I was not informed
- Other (please specify): _______

18. On a scale of 1 to 10, how clear was the information you received before the operation?
19. Have you ever felt that your ethnicity/origin influenced the care you received?
20. Before the operation, did you have a clear understanding of how your recovery would progress?
21. On a scale of 1 to 10, how did your recovery compare to your expectations beforehand?
22. Did you experience complications after the operation?
23. On a scale of 1 to 10, how clear was the information you received after the operation?
24. On a scale of 1 to 10, how satisfied are you with the communication with your healthcare providers?
25. Do you have any remarks, additions, or suggestions?

Standardized questions for collecting self-reported data on gender identity and race and ethnicity, endorsed by the Joint Commitment for Action on Inclusion and Diversity in Publishing

With which **gender** do you most identify? Please select **one** option:

- Woman
- Man
- Non-binary or gender diverse
- Self describe* *[opens text box]*
- Prefer not to disclose

*What are your **ethnic origins** or ancestry?*

Please select **ALL** the geographic areas from which your family's ancestors first originated:

- Western Europe (e.g. Greece, Sweden, United Kingdom)
- Eastern Europe (e.g. Hungary, Poland, Russia)
- North Africa (e.g. Egypt, Morocco, Sudan)
- Sub-Saharan Africa (e.g. Kenya, Nigeria, South Africa)
- West Asia / Middle East (e.g. Turkey, Iran, Saudi Arabia,)
- South and Southeast Asia (e.g. India, Indonesia, Singapore)
- East and Central Asia (e.g. China, Japan, Uzbekistan)
- Pacific / Oceania (e.g. Australia, Papua New Guinea, Fiji)
- North America (Canada, United States)
- Central America and Caribbean (e.g. Jamaica, Mexico, Panama)
- South America (e.g. Brazil, Chile, Colombia)

*How would you identify yourself in terms of **race**?*

Please select **ALL** the groups that apply to you:

- Asian or Pacific Islander
- Black
- Hispanic or Latino/a/x
- Indigenous (e.g. North American Indian Navajo, South American Indian Quechua, Aboriginal or Torres Strait Islander)
- Middle Eastern or North African
- White
